# How do public child healthcare professionals and primary school teachers identify and handle child abuse cases? A qualitative study

**DOI:** 10.1186/1471-2458-13-807

**Published:** 2013-09-05

**Authors:** Manuela WA Schols, Corine de Ruiter, Ferko G Öry

**Affiliations:** 1 Faculty of Psychology and Neuroscience, Maastricht University, Maastricht, The Netherlands; 2 TNO Quality of Life, Leiden, The Netherlands

**Keywords:** Child abuse, (Risk) detection, Reporting, Behavioral determinants, Teachers, Public child health professionals

## Abstract

**Background:**

Public child healthcare doctors and nurses, and primary school teachers play a pivotal role in the detection and reporting of child abuse, because they encounter almost all children in the population during their daily work. However, they report relatively few cases of suspected child abuse to child protective agencies. The aim of this qualitative study was to investigate Dutch frontline workers’ child abuse detection and reporting behaviors.

**Methods:**

Focus group interviews were held among 16 primary school teachers and 17 public health nurses and physicians. The interviews were audio recorded, transcribed, and thematically analyzed according to factors of the Integrated Change model, such as knowledge, attitude, self-efficacy, skills, social influences and barriers influencing detection and reporting of child abuse.

**Results:**

Findings showed that although both groups of professionals are aware of child abuse signs and risks, they are also lacking specific knowledge. The most salient differences between the two professional groups are related to attitude and (communication) skills.

**Conclusion:**

The results suggest that frontline workers are in need of supportive tools in the child abuse detection and reporting process. On the basis of our findings, directions for improvement of child abuse detection and reporting are discussed.

## Background

Child abuse represents a significant international public health problem [1]. In The Netherlands also, in 2010 the second national prevalence study of child maltreatment (NPM-2010) [2] showed that the annual prevalence of child abuse had remained the same compared to the first national prevalence study in 2005 (NPM 2005) [3] despite government policies aimed at improving detection and intervention (e.g., child abuse reporting guidelines) [4]. The NPM-2010 found 34 cases of child abuse per 1,000 children in the age range of 0–17 years [2]. Almost one third (31.4%) of the abused children were between 0 and 3 years old and 42.0% of the children were between 4 and 11 years [5]. In the United States, the Department of Health and Human Services (DHHS) reported similar findings showing that more than half of the abused children (54.5%) were age 7 or younger [6]. More specifically, they found that children in the age group 0–3 years comprised the largest proportion (30.4%) of substantiated child abuse cases. Children in the age group 4–7 years made up the second largest group (24.1%) of confirmed cases. In addition, young children are often the victim of more serious forms of child abuse; more than three-quarters (76.6%) of the children who died as a result of child abuse were under the age of 4 [6].

The Dutch (preventive) health service system for children from 0 to 19 years monitors approximately 97% of all children between ages 0 and 4 during home visits and visits at well-baby clinics. These services are widely used by Dutch parents, also by parents belonging to ethnic minorities. Embedded in its statutory task, this system provides several standard services (e.g., vaccination, preventive health checks) aimed at protecting and promoting children’s health [7]. Two weeks after a child is born, the public child healthcare nurse conducts a standard home visit. If necessary, additional home visits can be scheduled. The provision of treatment is not included in the basic preventive package, but public child healthcare workers can and do make referrals [8].

All children living in The Netherlands are obligated by law to attend school from age 5 as required in the Primary Education Act (“Wet op het Basisonderwijs”; effective as of August 1, 1985). Thus, Dutch frontline professionals such as primary school teachers and public child healthcare workers see almost all children aged between 0 and 11 years, who are the most vulnerable to become a victim of child abuse. Due to their coverage of children, primary school teachers [9] and public child healthcare workers [10] play a pivotal role in the prevention and identification of child abuse among children most at risk.

To reduce the prevalence of child abuse and its related costs and human suffering, different preventive strategies can be used, from primary to secondary to tertiary prevention [11]. In the present study, we will focus on primary and secondary prevention. Primary prevention includes the detection of risk factors for child abuse and the detection of cases of child abuse. For primary prevention, frontline workers need to become *cognizant* of child abuse risk factors such as domestic violence [12], parental mental health problems [13,14] and poor parenting skills [15], and stimulate parents to seek preventive help. Secondary prevention is targeted at specific “at risk” adult and child populations. Frontline workers can use several strategies related to secondary prevention, such as monitoring the child, making referrals to (mental) health services [16] and reporting to child protection services.

It is generally acknowledged that the identified and reported cases of child abuse are only a small representation of the actual cases. Research shows that the underreporting of child abuse by public child healthcare nurses [17,18] and primary school and kindergarten teachers [19-21] is still rather common. Feng et al. found in a sample of 598 Taiwanese kindergarten teachers that *“A total of 66 (11%) kindergarten teachers indicated that they had suspected at least one incident of child abuse but did not report the case”* (p.126) [21]. In addition, Goebbels et al. concluded on the basis of a study of 296 elementary school teachers that two-thirds of the non-reporters, i.e., teachers who indicated that they had ever failed to report a suspected case of Child Abuse and Neglect (CAN), had failed to report in more than one such case [19]. With regard to the detection of child abuse, Reijneveld et al. found that the detection of suspected physical and sexual abuse by public child healthcare workers had not increased over a period of 5 years (1996/1997-2002/2003), despite new regulations and/or good intentions [8]. It is hypothesized that these frontline professionals experience a number of obstacles that prevent them from detecting and reporting suspected child abuse cases adequately.

The present study aimed to investigate Dutch public child healthcare professionals’ (e.g., nurses and physicians) and primary school teachers’ child abuse detection and reporting behaviors and attempt to formulate directions for supporting behavioral changes. To facilitate the investigation of this complex set of behaviors, we used a theoretical framework, the Integrated Change (I-Change) model. This model describes factors (e.g., knowledge, attitude and skills) that are involved in any behavioral (change) process [22]. The model stems from the health promotion field and comprises concepts from the Theory of Planned Behavior [23], Social Cognitive Theory [24], the Transtheoretical Model [25], the Health Belief Model [26], and Implementation and Goal Setting Theories [27]. Behavioral change processes are described as consisting of three phases: awareness, motivation, and action. The I-Change model hypothesizes that the more abilities and skills a person possesses, the more likely intentions will be turned into action. Personal and environmental barriers may negatively impact individuals’ intentions, making it less likely that the intention is followed by the desired behavior. A person’s intention is assumed to be directly influenced by motivational factors such as social influences, self-efficacy, and attitude. The latter refers to perceived benefits and disadvantages of the behavior in question. Self-efficacy refers to an individual’s perception regarding his ability to carry out the preferred behavior [28]. Social influences are related to perceived norms of important others with regard to the behavior (social norms) and the degree of support a person receives for carrying out the behavior (social support). The model assumes that these motivational factors are determined by a number of distal factors, including awareness (e.g., knowledge, risk perception, and cues to action), information, and predisposing factors [22]. At the time the present study was conducted, child abuse legislation in The Netherlands differed from that in many other countries. Unlike other countries (i.e., Australia, United States, Canada, and Taiwan) that have established mandatory reporting laws [29], in The Netherlands, such a law has not been enacted and professionals working with children have no legal obligation to report (suspected) child abuse to the appropriate authorities. However, since 2002 there are national guidelines on reporting child abuse, comprising rules of conduct and instructions for both citizens and professionals when they suspect or identify a case of child abuse [4]. According to these guidelines, teachers are responsible for detecting abuse and make abuse a subject of discussion. However, they are not responsible for acting upon signs of abuse. The latter is the task of school social workers, school psychologists, the school principal or the school director [30]. For the preventive child healthcare system the guidelines suggest that every organization needs to employ a specific person with child abuse prevention in his/her portfolio (Dutch: ‘Aandachtsfunctionaris Kindermishandeling’). This person is responsible for the implementation of the guidelines [31].

We hypothesized that teachers and public child healthcare nurses and physicians detect and handle child abuse differently. Although public child healthcare nurses/physicians, and primary school teachers are active in frontline care and all have expertise in child development, these two types of professionals differ with regard to the frequency of their contact with children, total amount of time spent with the children, relationship to the child and its parents, the child’s age, and their legal position. In comparison to other professionals, teachers spend more time in daily contact with children and they can directly observe potential changes in the child’s appearance, behavior and progress, or unusual or uncharacteristic behavior that may be signs of abuse and neglect [32]. Teachers are also likely to develop a confidential relationship with children [33] and to be involved in children’s disclosures about maltreatment [34]. Teachers see children’s parents, for example, during standard parent-teacher meetings regarding the child’s development.

Public child healthcare nurses and doctors may observe signs of child abuse in the child’s behavior and appearance and in the family’s behavior, but they will also rely on knowledge acquisition from outside third parties [35]. Their contacts with the child and his/her parents are brief and infrequent. These professionals working in the preventive child healthcare system do have a statutory obligation to signal risk factors for child abuse and parental dysfunction as required by the Public Health Act (“Wet Publieke Gezondheid”; effective as of December 1, 2008), while this is not a core task of teachers. We expected that public healthcare workers would express more positive attitudes toward child abuse *detection* and *reporting* compared to teachers, as a result of their statutory task to protect the welfare of children. Due to the absence of a legal obligation to *report* child abuse, we hypothesized that neither teachers nor public child healthcare workers would see it as their core task to *report* suspected cases of child abuse. Thus, the goal of the current study was to investigate frontline professionals’ experiences with child abuse detecting and reporting, with a particular emphasis on the factors that may impact suboptimal detection and reporting.

## Methods

### Participants

The study was conducted at two Dutch local health services organizations; ‘Icare’ in the Province of Drenthe and ‘Groenekruis Domicura’ in the Province of Limburg. Also, three primary schools across The Netherlands were involved in the study. In 2009, the median disposable income for all households of the three included municipalities (i.e., Maastricht, Meppel and Assen) was 22,500, 28,100, and 27,200 euro’s respectively. The median disposable income for all households in the Netherlands was 28,200 euro’s [36]. With regard to ethnic composition, 27,150 (22.9%), 3,269 (10.2%), and 9,268 (14%) people, respectively of the people living in the included municipalities were migrants. In 2009, in total 3,287.706 migrants were living in The Netherlands. This means that the total percentage of migrants in the Dutch population was 19.9% [37]. Thus, all municipalities in which we conducted our study had a somewhat lower average income and two of the municipalities had a lower than average representation of migrants compared to the overall Dutch population. Organizations were recruited through personal connections. We asked approval from the principal of each school to contact individual teachers for participation in our study. In addition, approval was obtained from Icare’s management and a Domicura professional with child abuse prevention in his/her portfolio to approach nurses and physicians for participation in our study. Participation for both primary school teachers and public child healthcare workers was voluntary. Participants were public child healthcare nurses (*n* = 11), public child healthcare physicians (*n* = 6), primary school teachers (*n* = 15) and a school principal (*n* = 1). All participating public child healthcare workers were female. Nurses’ average age was 44.5 years (SD = 9.1; range = 30–58) and their average work experience was 15.6 years (SD = 5.5; range = 7–25). Public child healthcare physicians had an average age of 45.7 years (SD = 6.3; range = 33–50) and 15.6 years of work experience (SD = 7.5; range = 2.5-23). The group of primary school teachers consisted of 13 female and 3 male participants, including one school principal with an average of 21.3 years of work experience (SD = 12.9; range = 2–40). Their average age was 43.3 years (SD = 12.6; range = 24–59). Some of the teachers were also employed as a remedial teacher. The groups did not differ significantly with regard to age and years of work experience.

### Procedure

Participants were recruited through an announcement via e-mail or a hard copy leaflet, distributed at the participating schools and the local health services organizations, stating that researchers were looking for teachers and public child healthcare workers who wanted to talk about their experiences with child abuse cases they encounter in their daily work. The announcement explained that the results would be used in the development of a child abuse detection tool. During the years 2010–2011, the Mini-CARE method was developed and implemented in the preventive child healthcare in the Province of Flevoland. The findings of a validation study of this tool are forthcoming (Schols, de Ruiter, Broers, Kok: Early detection of child abuse risk by community nurses: Development and initial validation of a questionnaire for parents and a nurse-rated tool, in preparation). Written informed consent was obtained from all study participants. All were assured of confidentiality for themselves and their clients (children and families) discussed during the interview. Ethical approval for this study was obtained from the Ethical Committee of Psychology of Maastricht University (number ECP-81 14-04-2009-2).

The choice for the focus group interview method was based on the topic; the handling of (suspicions of) child abuse in daily work. Focus groups are advantageous, because they make it possible to gain insight into the sources of complex motivations and behaviours [38]. In addition, focus groups are a useful data collection method for investigating social norms as well as the diversity of perspectives of subgroups on a specific topic [39]. Moreover, participants can react and discuss topics and provide comments on each other’s statements. Focus group interviews were conducted based on participants’ profession; primary school personnel (i.e., teachers and school principal) and public child healthcare professionals (i.e., nurses and physicians). Thus, the focus groups were as homogeneous as possible and therefore it was possible to explore the opinions and experiences of the subgroups under investigation.

Groups comprised of 5 to 6 participants. In total, six focus group interviews were held; three among primary school personnel and three among public child healthcare professionals. The average duration of the interviews was 90 minutes (range = 78–112 minutes). Two trained interviewers led the focus group discussions at the public child healthcare workers’ workplaces and at the primary schools. A set of interview topics was used to guide the discussion. Interview topics were derived from the existing research literature on child abuse detection and reporting. Topics were divided into three main topics: signs of child abuse, the value of a child abuse (risk) detection tool and dealing with signs of child abuse (e.g., responsibility issues with regard to detecting and reporting of child abuse; see Additional file 1 for the interview questions). Open ended questions allowed participants to speak freely about their opinions, attitudes and experiences. The first author of this article was present at all focused group meetings. All interviews were audio-recorded and transcribed verbatim.

### Data analysis

After transcription, the researchers reviewed the interviews several times. Themes and issues that were present in the interviews were discussed. An in-depth thematic analysis to identify key issues was undertaken by the first author, which means that she independently developed a codebook based on the themes and issues that arose in the interviews. The I-Change model was used as a theoretical framework in identifying key issues. Emphasis was placed upon the subjective perspectives of the teachers and public child healthcare workers, because we were interested in their experiences with the detection and reporting of child abuse. Areas of consensus and disagreement between individual participants and between the two professional groups were particularly highlighted. Then, the conceptualisation of the themes was discussed with the second author. In case of disagreement, consensus between the researchers was reached by discussion. The final step in the data analysis concerned the selection of representative quotations to illustrate the views and attitudes stated by the participants. All quotes were translated from Dutch by the first author. During this process, the focus was on retaining the verbatim character of the original statement.

## Results

### Primary school teachers

#### Awareness stage

According to the I-Change model, the awareness stage is influenced by several information factors, such as the *source* of information and *predisposing factors* (e.g., cultural factors), and arises through the *level of knowledge*, the presence of *cues to action* and the *perception of risk*[22]. Table 1 provides an overview of the results as related to the awareness stage of the I-Change model.

**Table 1:** Determinants of awareness of professionals, as categorized by the I-Change model

		**Teachers**	**Nurses and physicians**
**Determinants I-Change model**	***Key themes***	***Sub themes***	***Sub themes***
Cues to action	Actual action cues	Signs of abuse	Signs of abuse
		● Various sources of abuse signs	● Various sources of abuse signs
		● Child is an important source in child abuse detection	● Preschool is an important source in child abuse detection
	Intuition	● Starting point of child abuse detection	● Starting point for systematic investigation
		● Precedes recognition of actual signs	
	Investigation strategies for confirming action cues (→ See Table 3; Action plans)	● Observing parents, child, and parent–child interaction	● Direct observation of parents and child
			● Building relationship with parents
		● Building relationship with parents	
			● Collecting collateral information
		● Talking to parents and/or child	
			● Conducting home visits
			● Monitoring and registration
Knowledge	Knowledgeable	● Definition of child abuse	● Definition of child abuse
		● Different types of abuse	● Different types of abuse
	Lack of knowledge	● Theoretical and practical knowledge	● Theoretical and practical knowledge
		● Lack of education	
		● Need for (more) high quality education programs	
Risk perception	Underestimation risk of neglect	● Subjectivity of norms and values	● Subjectivity of norms and values
		● Identification with parents	
		● Other justifications for underestimating abuse risk	

##### Cues to action

The results show that teachers become aware of alleged child abuse cases through different cues to action, such as actual signs of child abuse (e.g., bruises) or worrying family situations (e.g., divorce) from different *sources*, including the child, the child’s parents, other children’s parents, the police, the child’s friends, former schools the child has attended, neighbours, colleagues, local network, and school social workers. Teachers indicated that the child’s behaviour plays an important role in the awareness process of (possible) cases of child abuse. One teacher noted: *“Some children are very withdrawn, very anxious, or very present in the foreground. These behaviours differ per child, but it is behaviour that stands out or behaviour that does not feel right”*. In specific, sudden changes in the child’s behaviour or remarkable behaviour could serve as an indicator as well. Another teacher said: *“If a child suddenly shows a huge behavioural change or suddenly becomes hyperactive, then our feelers should get activated. This doesn’t immediately mean that child abuse is present, but it could be an indication. I do think it is important that we are attentive to these signs”*. However, teachers also stated that not all abused children display signs of abuse. An explanation for this could be that children experience the school as a safe place or due to “super-coping” of the child. The latter means that the child has found a way to cope with the adverse home situation or to compensate for parental deficits and has therefore become self-sufficient. In addition, teachers stated that children’s loyalty to their parents makes it difficult to detect child abuse. Teachers argued that especially in case the child does not display any signs of abuse, information from the other sources mentioned above, such as police and previous schools, becomes extremely valuable for detecting child abuse. The local care network, including public child healthcare workers, other teachers, the police, is also of great importance, since teachers acknowledged that they fail to see signs of abuse: *“I sometimes think: Am I not looking carefully?”* Neighbourhood or local police officers who spend most of their working hours in an assigned district were seen as a valuable source, because they know a lot about the families living in their district. In addition, teachers stated that they take action cues from the police more seriously than, for example, a child’s statement of abuse. Teachers argued that children sometimes make up things. Therefore, teachers are more hesitant to act upon the latter signs of abuse, because they are afraid to falsely accuse parents (false positives). This also leads to teachers’ caution with regard to interpreting signs of abuse: *“In case a child does not want to be touched, you have to be careful putting a label on it immediately”*.

Besides the identification of actual signs of abuse and worrisome family situations, teachers indicated that their gut feeling that something is wrong with the child - so-called intuition - was another *cue to action* and was viewed as the starting point of child abuse detection. More specifically, this feeling often precedes the recognition of actual signs of abuse and activates teachers to investigate the source of their feeling of discomfort. Teachers argued that intuition develops through someone’s previous experiences: “*I have learned over the past few years to pay more attention to the feeling that something is wrong with a child than to bruises on a child’s body. Subsequently, you have to investigate what causes this feeling of discomfort*”. Investigation strategies teachers mentioned included: observing the parents, the child, and the parent–child interaction, and talking to the parents and/or the child. Teachers reasoned that observing the parent–child interaction is possible only in young children, because when children become older their parents do not bring them to school anymore. Investment in the relationship with parents was also seen as an important strategy in the detection phase of child abuse and to motivate parents to accept help.

##### Level of knowledge

Knowledge is regarded as an important factor for the identification of child abuse and comprises knowledge of, for instance, the definition and baseline rates of child abuse, signs of child abuse, and child abuse reporting procedures. Teachers were *cognizant* of the different types of child abuse; physical abuse, emotional abuse (e.g., being witness to domestic violence, overprotection), physical neglect (e.g., no proper clothes, inadequate food), emotional neglect (e.g., lack of attention; discontinuity of care), and sexual abuse. In addition, teachers viewed the repetitiveness of the abusive behaviour as a core characteristic of child abuse. Teachers considered the definition of child abuse not as an unambiguous fact. They indicated that in the decision process of what constitutes child abuse and whether or not child abuse is present, social and cultural factors, such as their own upbringing, complicate this process. Some teachers expressed that they would like to gain more knowledge in the areas of child neglect and child emotional abuse, perhaps because these forms are less obvious.

However, teachers were experiencing a lack of knowledge of signs of abuse, baseline rates of abuse, and child abuse reporting procedures. Although teachers indicated to become aware of child abuse by paying attention to the child’s behaviour, one teacher illustrated a child sexual abuse case in which signs of abuse were not interpreted correctly and/or linked to the possibility of abuse. She reported in an emotional voice: *“I have to admit honestly that in this case I probably have been very naïve. I never thought of child sexual abuse. I still think about this case very often. That I did not think of sexual abuse. I just thought that it was a badly raised child and it did not cross my mind that there could be something really wrong”.* Awareness of the baseline rates of child abuse is lacking as well. This was demonstrated by the following remark of a teacher: *“I would be disappointed if we are suddenly specialized in identifying child abuse, because that would imply that child abuse is very common”.* Since statistics show that in The Netherlands in every classroom probably at least one child is a victim of child abuse, this illustrates some teachers’ lack of knowledge regarding base rates. However, this lack of awareness was not true for all teachers. One remedial teacher admitted that he obviously must have missed cases of child abuse during his daily work. In addition to theoretical knowledge of child abuse, practical knowledge also plays an important role in the child abuse reporting process. Practical knowledge refers to knowledge acquired through previous experience. Teachers indicated that this type of knowledge is important because there is a lack of attention to child abuse issues, including the practice of communication skills, in their education. Teachers stated that available post-graduate education programs on child abuse did not always address their particular needs. In their opinion, examples used during these training programs often do not reflect reality. According to one teacher, the awareness of child abuse could be increased by using video recordings, inviting someone who was a victim of child abuse and an expert with ample professional experience in handling alleged cases of child abuse.

##### Risk perception

Regarding risk perception, some teachers stated that they do not always want to fully acknowledge the seriousness of a child’s situation. To justify the situation, it seems that they try to make up excuses for the parents as well for themselves: *“You can only do something when the child is in your class”*. Teachers also have a tendency to identify with parents who have limited financial means: *“The parents themselves might not perceive it as abuse, because they are doing their best and maybe they just cannot do better”*. Thus, teachers might tend to underestimate the risk of neglect. One teacher exemplified: *“This was a case in which some teachers thought that neglect was present. Then we also visited the mother at home and she also indicated that it was all difficult for her, but how she was able to cope with the situation of having seven children as a single mother”*. Another teacher responded to this remark: *“And if she does this in a very loving way, than this situation might be better than say a lawyer who is verbally aggressive towards his child all the time”*. Teachers tend to hide behind norms and values which they apply differently to different people and which are not objective. They use this ambiguity as a justification to not act upon perceived signs.

#### Motivation stage

The I-Change model hypothesizes that a person’s intention to perform a specific behaviour is influenced by several motivational factors such as *attitude*, *social influences*, and *efficacy*. Table 2 describes the results of this study as related to the motivation stage of the I-Change model.

**Table 2:** Determinants of motivation of professionals, as categorized by the I-Change model

**Determinants I-Change model**	***Key themes***	***Sub themes***	***Sub themes***
Attitude	Responsibility	● Feeling responsible for detecting signs	● Feeling responsible for monitoring, motivating parents, and reporting
		● Not feeling responsible for reporting child abuse	
	Negative aspects	● Negative outcomes of reporting for child, family and teacher	● Dissatisfaction with quality, speed and continuity of care
Social influences	Support systems	● Internal and external sources of support	● Internal and external sources of support
		● Direct colleagues largest source of support	● Direct colleagues largest internal support system
			● Preschools important source of external support
	Types of support	● Emotional support	● Emotional support
		● Support in detecting and checking signs	● Support in checking signs
		● Talking to parents about suspicions of abuse	
	Lack of support	● Need for formal embedding of child abuse issues in the school system	● Little resources for addressing child abuse at the organizational level
		● Child abuse reporting agencies and care providers	● Child abuse reporting agencies
Self-efficacy	Low self-efficacy (See Table 3; Performance skills)	● Talking to (highly educated) parents	● Talking to (highly educated) parents
		● Motivating (highly educated) parents	● Detecting emotional abuse
		● Interpreting signs	
		● Talking to children	
		● Communication tool makes it safer to talk to parents	

##### Attitude

Although most teachers see it as their job to detect signs of child abuse, not all teachers agree upon their responsibility to act upon these signs and have the intention to report suspected child abuse cases to child protection services. For Dutch teachers it is quite unusual to report child abuse cases to child protection [30]. Mostly, teachers report their suspicions of child abuse cases to the principal of their school. One teacher said: *“If we get more responsibilities in this, we will get deeper into this and I am not sure how I would handle this. Then, I will take it home, but this will not make me happier”*.

Teachers reported several experiences of reporting suspected cases of child abuse and agreed that the result of reporting was not always positive for the child and its family. Teachers were lacking confidence in follow-up care for parents and the child. One teacher gave an example of professionals’ reluctance to act: *“…child welfare agency. These professionals often hide behind the excuse that parents do not have a request for help. We think that the child is the request for help”*. Thus, the results imply that not only teachers are hesitant to act upon their suspicions, but that also child abuse reporting centres do not act adequately. Teachers’ distrust in child welfare is fueled by the presence of waiting lists, discontinuity of care, bureaucracy, vague communication, lack of follow up, and the delayed start of actual care. Teachers’ frustration about failing care is exemplified by the next quote: *“The child’s condition becomes worse. You see that child. That child slips through your fingers. You can hear the child screaming for help, but you cannot do anything”.*

With regard to the consequences of reporting child abuse, some teachers argued that the situation of the child after the report may even become worse, for example, parents might move to another region as an escape or the foster care offered is not adequate. Teachers suggested that there should be one organization that bears responsibility for the care of the child and its family, and the teacher should be kept informed. Teachers stated that a report to child protection could not only result in negative consequences for the child, but also for themselves: parents may blame the teacher and can become aggressive: *“It was reported once by the school and then a teacher and I (remedial teacher) got blamed for this. At that time, it went well for a year and when it went wrong again we had to discuss it with the parents yet again”.*

##### Social influences

A person’s motivation is also affected by *social influences*, including the support an individual receives in carrying out the behaviour. We asked teachers in what way they receive support in the child abuse reporting process. Several support systems, including internal and external sources of support, were mentioned. Internal sources of support included the school principal, colleagues, the school social worker, and remedial teachers. Direct colleagues were regarded as the largest source of support. This support included emotional support, support in detecting and checking signs of abuse, and in talking to parents about suspicions of child abuse: “*It is especially nice if you have two teachers per class, because then you are able to test your view and to discuss this view with your colleague. You can also check your view with the remedial teacher of the school or in the entire school team. Or you can ask the previous teacher about the child’s behaviour. If the specific behaviour was not present in the past this could reinforce your observation that something is wrong”.* With regard to organizational support, teachers expressed a need for formal embedding of the handling of child abuse in the school system: *“That we do not pay attention to this phenomenon only this year, but that we structurally address this phenomenon: a) we will keep our knowledge up to date and b) the child abuse detection tools are part of the teaching resources”.*

Teachers reported that their experiences with the official child abuse reporting agencies, an external source of support, were both positive and negative, depending on the contact they had. One remedial teacher said: *“Very positive and very negative. Positive experiences relate to the person at the other end of the phone. We have had a person working at the child abuse reporting agency who was very committed and who acted instantly which was officially not allowed, but we also had people who acted according to the rules and said: I will consider it and you will hear from us again. Consequently, you do not hear from them anymore”.* Experiences with other care providers were not always positive either. Teachers expressed they sometimes did not feel taken seriously by care providers. Other external sources included local policemen, the school doctor, and the local care network. The local care network lowers the threshold to contact professionals from this network, because teachers already knew them from previous network meetings.

##### Self-efficacy

Concerning *self-efficacy*, which refers to an individual’s perception of his/her ability to carry out the behaviour in question, the results showed that teachers do not feel confident about communicating with parents, talking to children, and detection and interpretation of signs of abuse. Teachers argued that children’s loyalty to their parents makes it difficult to talk to children about suspicions of abuse. With regard to concerns about the well-being of the child, teachers have difficulties confronting parents with these concerns: *“I had a conversation with divorced parents. They came together to the school for a parent-teacher meeting. So I said very positively: ‘I am glad that you came together’. But it was not going well with this girl, because this girl was arriving in tears at home or at school on Mondays. So, I expressed this concern to the parents and asked them: ‘What can we do about this?’ The next thing that happened was that parents got into an argument right in front of me. Then, I asked myself: What am I going to do with this? You just do not know what to do”*. In particular, teachers find it difficult to deal with highly educated and assertive parents and with parents who tend to lie; they find it difficult to motivate these parents. Teachers hoped that a child abuse communication or detection tool would help them be more objective and neutral in their conversation with parents and that such an instrument would make it safer for them to talk to parents about their concerns.

#### Action stage

The final stage of the child abuse reporting process is the action stage. According to the I-Change model, positive intentions with regard to a particular behaviour do not automatically lead to the desired behaviour. Additional factors, which determine the onset of behaviour, are action planning, goal setting, and performance skills. See Table 3 for an overview of the thematic analysis of concepts related to the action stage of the I-Change model.

**Table 3:** Determinants of action of professionals, as categorized by the I-Change model

		**Teachers**	**Nurses and physicians**
**Determinants I-Change model**	***Key themes***	***Sub themes***	***Sub themes***
Performance skills	Lack of communication skills	● Communicating bad news to parents	● Clarification and communication of gut feeling
		● Involving children in conversation	● Handling unmotivated parents
			● Dealing with parents who display aggressive behaviour
Action plans	Lack of strategy for acting upon signs of abuse	● Need for consultation in case of suspicions	● Need for direct consultation with colleagues during the complete process
		● Need of clear guidelines with regard to procedures	
Barriers	Internal barriers	● Teachers’ feelings of guilt towards children	● Relationship with parents creates a blind spot
		● Fear of parents’ reaction to the report	● Maintaining relationship with parents
		● Fear of false positives	● Personal fears (for asking sensitive questions)
External barriers		● Privacy laws	● Safety risks for the professional
		● Parents’ control in accepting help	● Small distance between nurses’ work district and their home
		● Impossibility to report anonymously	

##### Performance skills

When teachers were asked what they needed in order to deal more effectively with child abuse in their daily work, they clearly expressed their need for practice in communication skills. Video recordings of practice conversations could have added value. Teachers indicated that they have difficulty finding the right words in their dialogue with parents: *“Sometimes you hear something from someone else and you think: ‘I should have had this sentence in my mind’”.* In addition, they are in need of directions to deliver a bad news message and how to motivate parents who don’t see anything wrong. Teachers expressed they also want to learn to discuss child abuse with children. This is important for teachers, because they have the abused child in their class, which implies that teachers’ care for the child does not stop after making the report.

##### Action planning

With regard to *action planning*, teachers mentioned that they are in need of clear guidelines with regard to procedures, steps that need to be taken, and persons with whom concerns can be discussed. Consultation with either child abuse reporting agencies, other child welfare agencies or a competent social worker could be supportive as well. Within this context, competency refers to having knowledge of the local social infrastructure map, because this is changing continuously, according to teachers.

##### Barriers

Teachers reported several *barriers* which heighten the threshold for child abuse reporting, including feelings of guilt towards the children, the impossibility to report abuse anonymously according to Dutch statutory law, fear of parents’ reaction to the report, parents’ control (within the voluntary scope of accepting help), privacy laws, and fear of false positives. Parents’ control becomes an issue when parents are unmotivated, because parents have to give permission for the start of help. Due to their daily contact with children and their specific position, teachers often struggle with the dilemma how long they have to try to collaborate with parents in case of worries. An example was described by one of the teachers: *“I asked parents why they let their four-year old child go to school alone by bike along the water. I will call the parents when the child is 5 minutes late, but who knows by then we might already have been 15 minutes too late”*. Feelings of guilt relate to situations where children are taken from the home. Teachers explained how the impossibility to report anonymously can form a barrier: *“But if you have the child in your class, then it is most likely that you were the one that made the report to the school’s principal. That is how people think”.* In addition, they experience the impossibility to report anonymously as a barrier, because they have to inform the parents that they are going to make a report.

### Public child healthcare workers

#### Awareness stage

Table 1 gives an overview of the results as related to the awareness stage of the I-Change model for the public child healthcare professionals.

##### Cues to action

With regard to child abuse risk factors, physicians mentioned that the presence of risk factors does not automatically mean abuse is currently taking or is going to take place in the future, and indicated this as a complicating factor in child abuse prevention. Therefore, not only the detection of child abuse, but the process (e.g., discussing the risks of certain behaviours with parents) starting with the detection of risk factors plays an important role in their work as well. According to the workers, signs of abuse can originate from different *sources*: the child (e.g., physical signs, behavioural signs), the child’s social environment, the child’s parents, other professionals (e.g., day-care workers, nursery school teachers), and volunteers involved in high risk families. An example of a child’s behaviour that could lead to suspicions was given by one of the nurses: *“An anxious child, but also a child who clambers onto everybody’s lap”*. However, professionals stated that not all abused children display signs: *“I had a case in which a baby did quite well, but the surrounding gave so much signs and facts that something was wrong”.* This can serve as a complicating factor in the child abuse detection process since several nurses tended to regard signs originating from the child as the most important *action cues*. Notwithstanding, another nurse decided on the basis of parental signs that steps needed to be taken; *“But that was not difficult. There were two young children in the family and there were problems with aggression and alcohol. Acting upon this was not difficult at that time. I just took that step, because this was not something that was going to solve itself”.* Some nurses argued that they became aware of abuse mostly through parental signs (e.g., alcohol and/or drug abuse; overburdening) and family factors (e.g., poverty, divorce, living conditions) rather than through signs from the child. Public child healthcare workers argued that preschool was an important source of information in the detection of abuse, because day-care workers see the children regularly. Nurses and physicians indicated that it is important to encourage parents to sign their child up for preschool facilities, not only because the child benefits from preschool, but also for early detection purposes. Public child healthcare workers emphasized the importance of regular contact with other care providers (collateral sources) aside from their contact with parents.

Besides the presence of actual cues to action, nurses refer to their intuition as an unconscious, automatic and important aspect in child abuse detection: *“It happens often to me that things happen in a flash and then I do not know what I have noticed specifically, but I do have this gut-feeling that something is wrong”*. In case nurses experience these intuitive feelings that something is wrong with the child, their next step is an attempt at objectifying these feelings. More specifically, professionals indicated that these intuitions often were the starting point for a systematic investigation which consisted of talking to parents, looking for evidence through direct observation and seeking collateral information. This is illustrated by the next citation: *“I saw a mother who was talking very satisfied about her child and how well he/she was doing. When I asked her questions about bottle-feeding she was not able to answer these consistently. The baby was not growing well and was looking pale. So, it starts with the gut-feeling that something is wrong”.* Professionals indicated that communication skills are very important in the relationship with parents; listening, asking the relevant questions, following through, and being respectful. Other strategies that were applied in case of suspicions of abuse were: conducting (unexpected) home visits, discussing the case with colleagues, investing in the relationship with parents, and monitoring and registration (i.e., keeping a file). The latter is exemplified by the next quotation: *“In this case we invited the mother regularly to the well-baby clinic and we looked how the baby was developing according to the growth-curve. We looked at the impression the child made, also in contact with others. And then we concluded: this is not going well”.* Concerning the registration of risk factors, one physician made the distinction between static (e.g., parents’ past experiences) and dynamic risk (and protective) factors for abuse and emphasized the importance of noting the dynamic risk factors, due to the changeability of these factors.

Nurses’ and physicians’ *knowledge* of child abuse is partly reflected in their definition of child abuse. They were *cognizant* of the different types of child abuse and defined child abuse broadly. One nurse said*: “All forms of lack of care for the child that should normally enable the child to maximally develop itself”*. Another illustration of defining child abuse broadly was provided by one of the participating physicians: *“I think that if the child doesn’t show signs of abuse, but if a lot of obstacles are present and nobody takes the responsibility to do something with these obstacles, then I think you are taking part in neglecting the child”*. They also made several other distinctions in defining abuse: passive/active, verbal/non-verbal, and conscious/unconscious. Although professionals argued that the presence of inflicted harm is an important aspect in defining child abuse, they agreed that the term child abuse is stigmatizing, because it implies the presence of an active, willful component while in reality the abusive parent is often merely incapable of good enough parenting.

##### Knowledge

With regard to *knowledge* of child abuse related issues (e.g., signs of abuse, baseline rates, and reporting laws), nurses indicated that they lack knowledge about normative psychosexual and psychosocial development of children. In addition, they indicated that they are not informed about children’s basic needs. One nurse exemplified: *“Is it bad when a child doesn’t get a warm meal for three years? Maybe a sandwich is sufficient as well”.* Professionals argued that guidelines concerning characteristics of good enough parenting would be helpful in detecting abuse. Professionals did seem to be aware of baseline rates of abuse: *“If I see the prevalence rates of child abuse, then I think I do not see these rates, so I have to miss signs/cases”.*

##### Risk perception

With regard to *risk perception*, one nurse argued that in case a child does not display any overt signs of abuse, the values of the professional might influence risk perception in the sense that values may lower standards of what is acceptable: *“…because otherwise I think this is a whole other environment than the environment I grew up in, but the child is actually quite happy”.* Workers seemed to use the subjectivity of their values as an excuse to not act upon signs. This was illustrated by the following quotation: *“But you also have to contend with other cultures in which children are treated very differently. So, if you mention the basic needs of a child, then what are these needs?”* Still, they showed some insight into this mechanism: “*We set the bar too low for what is still acceptable and too high for reporting”*. The results showed that workers tend to ignore particular signs of abuse, for example, an unhygienic environment. The perception of risk is also influenced by the number of signs; the more signs present, the higher the risk is rated: *“All together, it is an enumeration of circumstances/signs”.*

#### Motivation stage

Table 2 describes the results of this study as related to the motivation stage of the I-Change model.

##### Attitude

With regard to child healthcare professionals’ responsibility, the results showed that they regarded several tasks as falling within their responsibility, including motivating parents to accept help, monitoring the response to care, monitoring of high risk cases, and registration (keeping a file). They acknowledged their statutory responsibility to check whether a child’s needs as prescribed by the Universal Convention on the Rights of the Child [40] are met. Several physicians, although agreeing upon their statutory task, adhered to the view of a civil society [41] which means that everybody is responsible for the wellbeing of children, especially because children between the ages 4 to 19 are only seen three times by the preventive child healthcare system. Public child healthcare workers also try to assist other professionals (e.g., teachers) in becoming more competent in the domain of child abuse. One physician mentioned that they trained professionals working at preschool facilities how to communicate their observations to parents.

Professionals expressed their dissatisfaction with regard to the quality, speed, and continuity of care started at the request of public child health. With regard to speed, nurses argued that it takes much too long for care to start, and when care is started, it often ends too soon: *“At one moment I said to the case manager of the welfare agency: if you do not act upon it now, then I will report the case to the child abuse reporting agency”.* With regard to quality of care and contact with care providers, professionals mentioned that it is often not clear what the provided care entails. In addition, professionals indicated that they felt not always taken seriously by care providers and the child abuse reporting agencies, because they trivialize the problems, which reduces their willingness to report. This was illustrated by one worker: *“When I told them what the signs were, they said to me: we have much worse cases”*. Other complicating factors regarding contact with care providers consisted of poor coordination and difficulties with regard to the interchange of information: *“Everybody is busy detecting abuse and they do not know from each other who is doing what.”*

##### Social influences

Professionals’ response to the question how they receive support in the child abuse reporting process can be divided into internal and external support systems. Internal sources of support consisted of peer review (e.g., checking signs of abuse) and emotional support. Questioning each other with the help of solution-focused questions [42] was seen as an important part of peer review, because it stimulates further thinking. Other internal support systems mentioned were: evaluative meetings at the management level, guidelines/protocol, and the coordinator for the electronic child records. The latter focuses mainly on high risk families by monitoring and maintaining contact with the family. This can be regarded as a form of case management: *“It is mainly process control and at the time signs emerge and there is already care in the family, I am checking what everybody is doing, especially in case multiple care providers are present”*.

Although professionals mentioned that there is a specific colleague with child abuse in his/her portfolio, they stated that this colleague is often too remote from their daily practice with families and often too inexperienced. Several professionals also mentioned a lack of emotional support at the managerial level: *“I still go to my direct colleagues. At one moment when I had a case which I was distraught about I had to find help myself”*. Another aspect that was not taken up by the organization was the issue of safety. One nurse said that she arranged for herself that the police was on stand-by in a particular case in which she expected that a grandfather would come to the well-baby clinic and act aggressively. The public child health system also lacks the financial resources to allow scheduling of additional home visits, in cases of high-risk or serious concern. In some families continuous monitoring is needed to prevent abuse. One nurse exemplified: *“I have a case in mind which went well for a time, just because I visited them regularly”.* Professionals reported both positive and negative experiences with the official child abuse reporting agencies, an external source. Negative experiences were related to bureaucracy*: “This is not working. I am in need of discussing a case quickly by phone”*. Positive experiences consisted of follow-up contact with the agencies: *“They became more active in calling back and asking questions about the case: how is it going now?”* Other external sources of support included local police officers, preschool workers, general practitioners, school social workers, teachers, and mental health workers. One nurse exemplified the kind of support she received from the police by quoting: *“You can check with the police easily. Sometimes you do not know if this father is really aggressive or not. I check this sometimes before I visit the family. Is this safe or not?”* The results indicated that preschools were the largest external support system for the detection of child abuse.

##### Self-efficacy

With regard to *self-efficacy*, professionals indicated that they found it difficult to communicate with parents: *“I think we are just not able to have a difficult conversation with parents”.* More specifically, they explained that they find it difficult to deal with highly educated parents who are able to put on a friendly face. One physician indicated that she experienced difficulties checking if parents pay (enough) attention to the emotional well-being of their child and also in explaining to parents why this kind of support is important for a child’s development: *“But in case of a baby it is just difficult to explain that talking to the baby is very important, because otherwise the baby will not respond anymore”.* Nurses indicated that they would find an objective and transparent instrument helpful in motivating parents to seek care (and in detecting abuse): *“That you can show to the parents. This is proven. If these signs are present then this raises reason for concern. And these are not my personal norms and values”.*

Notwithstanding the above, professionals already react adequately in a number of situations, such as talking to parents about child abuse risk factors and their worries regarding a child’s situation. This is exemplified by one nurse in a conversation with an abused parent: *“If victims of child abuse become a parent themselves, feelings of the experienced childhood abuse can arise more easily and become more accessible”*. A different explanation came from another nurse: *“In times of distress the risk increases that you are going to act upon this in the same (abusive) way your parents did, even though this is not what you want”*. An illustration of discussing worries with parents in an adequate way was provided by one nurse: *“I do not use the term child abuse, but I try to start up help in the family. I also make it clear that I am worried about the situation”*.

#### Action stage

See Table 3 for an overview of the thematic analysis of concepts related to the action stage of the I-Change model.

##### Performance skills

When child healthcare professionals were asked what they required to deal more effectively with child abuse in their daily work, they stated that they would benefit from improving their communication skills by receiving training: *“To be able to detect child abuse I must be able to have a difficult conversation with parents”.* Nurses indicated they find it difficult to discuss with parents their gut feeling that something is amiss. They also expressed a need for learning how to communicate with parents who could become aggressive: *“I am thinking/looking where the door is. Am I safe here?”*. They also find it difficult to handle parents who do not want to cooperate or are resistant. They hoped a checklist would be helpful in sharing with parents concerns about abuse. However, they do not want a purely actuarial instrument; their professional judgment is important as well in the decision making process. Nurses argued that an instrument could also be helpful in communicating with care providers in the sense that their concerns would be taken more seriously. Furthermore, the instrument should be implemented broadly.

Besides training as a professional, one nurse stated that it also important to pay attention to the person behind the professional. Fears (e.g., asking sensitive questions), personal values and experiences around child abuse can act as an internal barrier and should be addressed in training and supervision.

##### Action planning

With regard to *action planning*, the results showed that professionals are in need of consultation with (direct) colleagues during the complete child abuse detection process and not only in reporting (alleged) abuse. It is important that consultation takes place promptly and not several weeks later. One nurse, who was acquainted with the child abuse reporting guidelines, expressed that the guidelines make it easier to have discussions with other professionals about specific children.

##### Barriers

Professionals noted several barriers in their daily work, both internal and external. The geographical distance between nurses’ work district and their home can act as a barrier to act upon signs of abuse, in case the distance is small: *“I experience this as a dilemma, because you are not only dealing with this particular family. If you are working in small villages, it spreads like wildfire, so you have to think about how you say and act upon things”.* Although the relationship with parents was mentioned as a tool for detecting child abuse and motivating parents to accept help, the relationship can also act as a barrier in the sense that it creates a blind spot in professionals which could lead to lowering their standards for good enough parenting. In addition, fear of losing contact with the parents could act as a barrier. Professionals were always balancing between watchful waiting on the one hand and taking action on the other hand: *“But then it is about not wanting to break off the relationship with parents. You want to do it together with the parents. But sometimes this is not possible anymore, but when is this point reached?”* Some professionals refer to safety risks as a barrier to talk to parents about suspicions of abuse and reporting abuse to child protection which could obviously lead to ignoring the most problematic cases.

## Discussion

In this study we performed focus group interviews with 33 frontline professionals working with children to investigate how these professionals deal with child abuse (issues) in their work. The validity of this qualitative study was increased by maximizing the richness of the data [43] by selecting experienced public child healthcare workers and primary school teachers carefully. Below we will discuss the findings in association with the three stages of the I-Change Model. In addition, the most salient differences between the two professional groups will be highlighted. Furthermore, recommendations for improvements are detailed.

At the awareness stage, the results showed few differences between the two professional groups. Both public child healthcare workers and teachers know about the different forms of child abuse. Their definition was consistent with the definition of child abuse in the empirical literature and in the extant Dutch Youth Welfare Law (2005; Article 1, sub m). Still, the results showed several gaps in professionals’ knowledge concerning child abuse issues. For teachers, the main gaps were related to base rates of child abuse, signs of abuse and reporting procedures, partly due to a lack of attention to child abuse issues in their teacher education. These results concur with a previous study of Feng et al. which showed that in a sample of 598 kindergarten teachers more than 81% reported that both pre-service and in-service child abuse education was inadequate [21]. Previous research found similar results for nurses [44,45]. Moreover, teachers seemed to have less knowledge of signs of abuse compared to public child healthcare workers; for example, the latter described the relation between risk factors and the onset of abuse in more detail. As a result of this, public child healthcare workers might be better at detecting child abuse risk factors and child abuse than teachers. An important shortcoming in nurses mentioned concerned their knowledge of children’s psychosexual development. These findings imply that pre-service education should address child abuse and child development much more thoroughly. Since professionals are more likely to report abuse when they are aware of the impact of the abuse on the child [46], training programs should emphasize the negative impact of abuse. However, increasing professionals’ knowledge will not be sufficient to improve child abuse detection and reporting, because other determinants of the I-Change model also play an important role in the decision process. For example, Goebbels et al. found that the availability of action plans with regard to child abuse detection and reporting also influenced teachers’ reporting behavior [19]. In addition, O’Toole et al. showed that post-graduate education alone did not predict child abuse detection and reporting [47].

At the motivation stage, both professional groups showed similarities with regard to perceived *support*; direct colleagues were regarded as the most important support system. Related to this finding, it is recommended that preventive child healthcare organizations reserve more resources for regular consultations among their workers. With regard to external sources of support, the (local) police was seen as an important support system, especially with regard to safety issues. The police was also considered important in the detection phase of abuse. However, the results also showed that police support was not addressed at the organizational level; safety seemed not to be a core topic on the management’s priority list. Roles and responsibilities of the involved parties need to be clarified. Our findings imply that statutory regulations alone do not offer enough support for professionals in the child abuse reporting process. For teachers, the local network was also an important source of support, due to the diversity of professionals contributing to this network. Since primary school teachers themselves do not report their suspicions of child abuse to child abuse reporting agencies, but to the school’s principal or remedial teacher, it is important that teachers, in case they suspect child abuse, feel supported by school management. Concerning *attitude*, the results showed that the public child healthcare workers view themselves as case manager and are more active in looking for signs of abuse compared to primary school teachers. The latter showed a higher threshold for acting upon suspicions of abuse. These findings may be due to differences in legal obligations of both professional groups. The health workers are also more comfortable talking to parents about suspicions of child abuse (*self-efficacy*). Both groups experienced difficulties in detecting emotional abuse and in explaining to parents that offering adequate stimulation to a child is important for the child’s development. Teaching aides for educating parents about children’s emotional needs and development are necessary. With regard to attitudes toward child abuse reporting, teachers were not convinced that the child would benefit from a report to child protection. This view is supported by findings from the recent Samson Report [48] of child abuse within Dutch youth care institutions. They found that children who were placed in out-of-home care were 2.5 times more likely to become a victim of child sexual abuse than Dutch children living with biological parents. For children with an intellectual disability this rate was even higher.

At the action stage, the two professional groups differed with regard to *skills*. Public child healthcare workers clearly possess more skills to communicate with parents compared to primary school teachers. Still, both groups indicated experiencing problems talking with highly educated parents. In addition, teachers also found it challenging to talk with children. This is a finding of concern, since teachers see children on a daily basis and of the two professional groups they have most opportunities to gain children’s confidence. These results imply that training in communication skills would be important for ameliorating the detection of abuse. Research has shown previously that mothers’ disclosure about her child’s psychological functioning to a physician predicts the detection of psychosocial problems [49]. Physicians’ interviewing style and communication skills are found to enlarge these disclosures [50]. *Barriers* for reporting that were identified by the participants were in line with the results of previous studies; being afraid of the consequences [51], feelings of guilt, not having confidence in follow-up care [52], and “feeling uncertain about the evidence” (p.342) [44]. The latter barrier could be addressed by using a structured risk screening instrument. Our results indicate that an instrument would be most helpful in cases in which signs of abuse are not obvious, because it would help with objectification.

Taking all findings into account, primary school teachers would most likely benefit from a program or method which involves both children and teachers and which is imbedded in the school‘s structure, because child abuse detection is not their primary task and therefore they often have limited time and resources to spend on child abuse issues. With regard to the design of a program, the use of interactive techniques such as active rehearsal and modelling is suggested, because literature shows that child sexual abuse prevention programs are most effective when interactive techniques are used [53]. The most effective personal safety programs comprise multiple sessions, opportunities to practice skills and parental involvement [54]. The results of the present study also imply that it might be favourable to employ two (part-time) teachers per classroom since they could discuss possible signs of child abuse. In addition, school support could serve as a motivational factor in the child abuse reporting process. The professional with child abuse prevention in his/her portfolio should be trained in assessing child abuse risk and communicating with parents and care providers of the family. They should also pass on their knowledge and skills to the teachers and assist teachers in managing a vulnerable child in the classroom. It is their task to monitor how the child is doing.

For the public health nurses, home visits are an important tool in the detection of child abuse (and child abuse risk factors) and subsequently in child abuse prevention. Olds et al. found that prenatal and early infancy home visits, in comparison to routine community care, led to improved pregnancy and postnatal outcomes for mothers and children [55,56]. A study into the long-term consequences of these home visits revealed that over a follow up of 15 years, the number of proven child abuse reports among nurse-visited mothers was almost half that of the not nurse-visited women. This effect was even larger among families who were more at risk [57]. Nurses in our sample acknowledged the benefits of home visits as well. In The Netherlands, several standard home visits are conducted in the first years of a child’s life. Furthermore, a child abuse risk detection tool should be imbedded in the home visiting system. Grietens et al. developed a scale for home-visiting nurses for identifying risks of physical abuse and neglect in mothers with a newborn child, the Early Risks of Physical Abuse and Neglect Scale (ERPANS) [58]. The ERPANS subscales “social isolation”, “distorted communication”, and “psychological problems’ were able to discriminate between non-abusive and recently abusive mothers [10]. Another effort to improve public child healthcare nurses’ risk assessment of parenting and child developmental problems was recently made by Staal et al. [59]. They developed a structured interview, the Structured Problem Analysis of Raising Kids (SPARK), using a three-step model; detection, clarification, and analysis. After the home visit the nurse completes the SPARK with an overall risk assessment. The SPARK was shown to possess discriminative validity; children from lower socio-economic status families scored significantly worse on the overall risk assessment compared to children from higher socio-economic status families [60]. The overall risk assessment of the SPARK was also shown to be predictive for child abuse reports to the official reporting agencies [61]. Aside from home visits, the public health nurses in our study also took on the role of case manager, by keeping in contact with care providers involved with at-risk families, to monitor the child’s wellbeing.

An inherent limitation of qualitative research is related to the generalizability and ecological validity of the study findings. Since we included nurses who were willing to participate in the study voluntarily, selection bias might have occurred in the sense that the participants of our study could have been more motivated and interested in child abuse prevention than the average public child healthcare worker or teacher. Due to the explorative nature of this study, the results cannot be generalized to frontline workers from other professions. Concerning the generaliziblity of the results to similar professional groups in other countries, we have to be cautious as well. Statutory obligations regarding child abuse reporting differ across countries and these impact the child abuse detections and reporting process via factors such as knowledge, attitude, and support. On the basis of a qualitative study, we are not able to make inferences about the causal relationship between the different behavioral determinants. It is unknown how the behavior determinants interact. In order to obtain an overall perspective on child abuse detection and reporting practices, it is required that future research focuses on other stakeholders such as youth care providers, school principals and management teams. Finally, at the time of the interviews were conducted, schools differed with regard to the use of the child abuse reporting guidelines, because the guidelines were not yet a legal requirement, may have influenced our study findings.

In the Netherlands, the national child abuse reporting guideline is effective as of July 1, 2013. The guidelines comprise ‘rules’ regarding conduct and instructions for citizens and professionals when they suspect or identify a case of child abuse [62]. The guidelines distinguish five steps; gathering and documenting signs of abuse, consulting a colleague, talking to the parents, judging the severity of the situation, and acting upon the suspicions of abuse (e.g., arranging help, filing a report). There will be no mandatory reporting regarding child abuse. The child abuse reporting guidelines could not only be helpful in providing direction, but they can also provide support, for example, reducing teachers’ feelings of guilt in situations where the child is placed out of the home due to a report. However, it is evident from our study that the reporting guidelines alone will not be sufficient to solve all the problems professionals encounter in this domain.

## Conclusions

In conclusion, the results of the present study demonstrate the complexity and dynamics of the child abuse detection and reporting process. Understanding of this process is crucial with regard to the development of an effective child abuse (risk) detection method. Ameliorating teachers’ pre-service education with regard to child abuse issues would be an important first step. Education programs, including pre-service education, for both professional groups should focus on the practice of communication skills. Child abuse reporting guidelines could increase professionals’ confidence to report in specific circumstances. Both professional groups are in need of methods for objectifying their suspicions, especially in case of circumstances in which implicit rather than obvious signs of abuse are present. The use of a structured method could strengthen the basis of their suspicions and could improve the detection and reporting of abuse.

## Competing interests

This research was supported by grants from ZonMw, the Netherlands organization for health research and development (project number 61300035), Stichting *Kinderpostzegels Nederland*, and Achmea Foundation Victims and Society (*Stichting Achmea Slachtoffer* &*Samenleving*) to Corine de Ruiter, PhD. There is no competing interests.

## Authors’ contributions

MWAS carried out the focus group interviews and drafted the manuscript. CR contributed substantially to the conceptualisation and design of the study and provided extensive feedback on the manuscript. FGÖ carried out the focus group interviews and provided feedback on the manuscript. All authors read and approved the final manuscript.

## Pre-publication history

The pre-publication history for this paper can be accessed here:

http://www.biomedcentral.com/1471-2458/13/807/prepub

## Supplementary Material

Additional file 1 Protocol for focused group interviews. Click here for file
