# Supplementary material for: How do public child healthcare professionals and primary school teachers identify and handle child abuse cases? A qualitative study
Source: BMC Public Health. 2013 Sep 5;13:807. doi: 10.1186/1471-2458-13-807 (PMC3847190; doi:10.1186/1471-2458-13-807)
Supplement: Additional file 1 — Protocol for focused group interviews. [file 1471-2458-13-807-S1.pdf]

Additional file 1

Protocol for focused group Interviews

1. Signs of child abuse

a. How do you define child abuse and what are, according to you, signs of child abuse?

b. Have you ever encountered child abuse cases in your daily work? During the past two years, how often did you make a report of (alleged) child abuse?

c. Can you tell us more about this experience? Did you use a detection tool or a certain method? If yes, which method or tool did you use?

d. How do you discover child abuse signs in you daily work?

e. Do you receive support from the preventive child-healthcare organisation, in which you are employed, to detect child abuse? If yes, can you explain this or give an example?

f. Do you think you missed signs of child abuse in the past or miss signs of child abuse at this moment, during you day-to-day work?

2. Value of a child abuse (risk) detection tool

a. What do you need to detect child abuse earlier?

b. Do you think that a child abuse detection tool can help you in detecting child abuse earlier?

c. What are important elements of a child abuse (risk) detection tool?

3. Dealing with signs of child abuse

a. How do you handle signs of child abuse in your daily work practice?

b. Do you receive organizational support with regard to taking action in (alleged) cases of child abuse? If yes, in what way are you supported? If no, what do you need from the organisation?

c. What is your opinion about the child abuse reporting guidelines and the mandatory reporting guidelines?

d. Can you mention reasons for not reporting child abuse even though signals of child abuse are present?

e. What barriers do you encounter in child abuse reporting?

f. Do you feel responsible for reporting (alleged) child abuse cases? Who do you think is responsible for reporting child abuse?
